# Colloidal III–V Quantum Dot Photodiodes for Short‐Wave Infrared Photodetection

**DOI:** 10.1002/advs.202200844

**Published:** 2022-04-10

**Authors:** Jari Leemans, Vladimir Pejović, Epimitheas Georgitzikis, Matthias Minjauw, Abu Bakar Siddik, Yu‐Hao Deng, Yinghuan Kuang, Gunther Roelkens, Christophe Detavernier, Itai Lieberman, Paweł E. Malinowski, David Cheyns, Zeger Hens

**Affiliations:** ^1^ Physics and Chemistry of Nanostructures Ghent University Krijgslaan 281‐S3 Gent 9000 Belgium; ^2^ Imec vzw Kapeldreef 75 Leuven 3001 Belgium; ^3^ Department of Solid State Science Ghent University Krijgslaan 281‐S1 Gent 9000 Belgium; ^4^ Photonics Research Group Ghent University Technologiepark‐Zwijnaarde 126 Gent 9052 Belgium

**Keywords:** InAs quantum dots, infrared sensing, printed photonics

## Abstract

Short‐wave infrared (SWIR) image sensors based on colloidal quantum dots (QDs) are characterized by low cost, small pixel pitch, and spectral tunability. Adoption of QD‐SWIR imagers is, however, hampered by a reliance on restricted elements such as Pb and Hg. Here, QD photodiodes, the central element of a QD image sensor, made from non‐restricted In(As,P) QDs that operate at wavelengths up to 1400 nm are demonstrated. Three different In(As,P) QD batches that are made using a scalable, one‐size‐one‐batch reaction and feature a band‐edge absorption at 1140, 1270, and 1400 nm are implemented. These QDs are post‐processed to obtain In(As,P) nanocolloids stabilized by short‐chain ligands, from which semiconducting films of *n*‐In(As,P) are formed through spincoating. For all three sizes, sandwiching such films between *p*‐NiO as the hole transport layer and Nb:TiO_2_ as the electron transport layer yields In(As,P) QD photodiodes that exhibit best internal quantum efficiencies at the QD band gap of 46±5% and are sensitive for SWIR light up to 1400 nm.

## Graphical abstract

A complete process flow to form photodiode stacks sensitive for short‐wave infrared (SWIR) light based on non‐restricted In(As,P) quantum dots (QDs) is proposed. Films made of semiconducting *n*‐In(As,P) QDs inks, formulated through apolar/polar QD phase transfer, form a rectifying junction with *p*‐NiO that is photosensitive beyond 1400 nm. This result highlights the prospect of printable SWIR opto‐electronics based on InAs QDs.

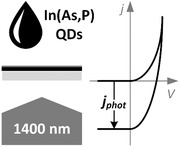

## 1 Introduction

Colloidal semiconductor nanocrystals or quantum dots (QDs) are an emerging opto‐electronic material that combines a suitability for solution‐based processing with widely tunable absorption and emission characteristics.^[^ ^1^, ^2^, ^3^, ^4^ ^]^ This dual asset spurred the development of a QD‐based opto‐electronic technology, where QDs stand out as visible‐light emitters for display and lighting and as infrared absorbers for solar energy conversion and sensing. Especially at short‐wave infrared (SWIR) wavelengths, that is, 1000 − 3000 nm, the possibility to directly print QD photodiodes (QDPDs) on silicon read‐out circuits makes for a unique path to create SWIR image sensors at far lower cost and smaller pixel pitch than current wafer‐bonding approaches.^[^ ^5^, ^6^, ^7^, ^8^, ^9^, ^10^, ^11^, ^12^, ^13^, ^14^, ^15^ ^]^ Current SWIR QDPDs consist of multilayer stacks that include PbS or HgTe QDs as the photo‐active layer. In line with the extensive work on single‐junction QD solar cells, charges are separated in such stacks either at the heterojunction formed between the QD film and one of the charge‐transport layers or through an internal *pn* junction formed within the QD film. PbS QDPDs with specific detectivities above 10^12^ Jones at 1550 and 1750 nm have been reported, while reports on HgTe QDPDs demonstrate photodiodes at even longer cut‐off wavelengths in the SWIR.^[^ ^15^, ^16^, ^17^, ^18^ ^]^

A significant issue hampering the adoption of QD‐SWIR imagers are the restrictions on hazardous substances, such as Cd, Hg, and Pb, issued by regulators worldwide. These directives have stimulated research in alternative QDs, where III–V QDs such as InAs and InSb are currently seen as the most relevant for SWIR applications.^[^ ^19^ ^]^ III–V semiconductors are widely used in opto‐electronics, also for SWIR applications, and in particular in the case of colloidal InAs QDs, major synthetic progress was made in the past 5 years. While initial synthesis methods to form colloidal InAs QDs were developed more than 20 years ago, the use of perilous and highly reactive precursors such as tris‐trimethylsilylarsine (TMS_3_As) limited research interest and mostly resulted in InAs QDs with an absorption edge of 1000 nm or less.^[^ ^20^, ^21^, ^22^ ^]^ The introduction of more gentle synthesis pathways based on trisdimethylaminoarsine (DMA_3_As) precursors gave new impetus to InAs QD research, with studies reporting band‐edge transitions up to 1400 nm, that is, well within the SWIR.^[^ ^23^, ^24^, ^25^, ^26^, ^27^ ^]^ Even so, only few studies report the formation of InAs‐based QDPDs, and published work invariably focuses on InAs QDs made from TMS_3_As that are active at wavelengths shorter than 1000 nm.^[^ ^28^, ^29^ ^]^

Here, we demonstrate the formation of QDPDs using three different sets of In(As,P) QDs made from DMA_3_As) and featuring a band‐edge transition at 1140, 1270, and 1400 nm, respectively. Leveraging recently published insight in the In(As,P) QD surface chemistry, we introduce a 2‐phase liquid–liquid extraction approach to replace the native, long‐chain oleylamine ligands by a combination of *n*‐butylamine and 3‐mercapto‐1,2‐propanediol. Films of such post‐processed In(As,P) QDs exhibit increasing conductivity at positive gate bias, indicative of *n*‐type doping; a result in line with ultraviolet photoelectron spectroscopy results (UPS) and previous literature studies. We therefore propose a QDPD stack where the *n*‐In(As,P) QD film is sandwiched between a *p*‐NiO hole transport layer and an electron transport layer made from Nb:TiO_2_ nanoparticles. These stacks all exhibit a dark current below 2 µA cm^−2^ at −1 V reverse bias, display an EQE spectrum that tracks the band‐edge absorption of the respective QDs, and show rise and fall times with a dominant time constant of ≈1 µs. This first demonstration of In(As,P) QDPDs that are active up to 1400 nm is a significant step in the development of QD‐SWIR sensors and imagers entirely based on non‐restricted compounds.

## 2 Results and Discussion

**Figure 1:**
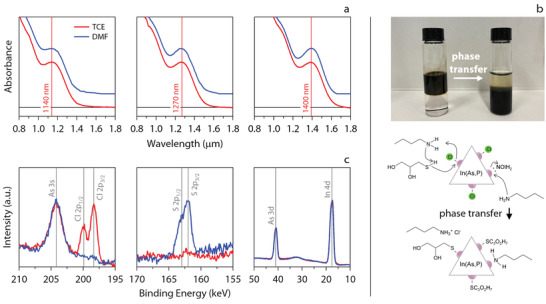
a) Normalized absorbance spectra of the three QD batches (red) measured in tetrachloroethylene (TCE) before and (blue) dimethylformamide (DMF) after phase transfer. For each set of spectra, the vertical line indicates the maximum absorbance of the band‐edge transition at 1140, 1270, and 1400 nm, respectively. The spectra after ligand exchange have been offset for clarity. b) (top) Photograph of the extraction of QDs from (top phase) octane to (bottom phase) DMF and (bottom) representation of the phase transfer chemistry when using 3‐mercapto‐1,2‐propanediol (MPD) and butylamine (*n*‐BuNH_2_) as phase transfer agents, indicating several reactions that bring about the replacement of the as‐synthesized ligand shell of chloride and oleylamine by deprotonated MPD and *n*‐BuNH_2_. c) X‐ray photoelectron spectra (red) before and (blue) after ligand exchange in different energy ranges, showing the disappearance of chloride, the appearance of sulfide and the preservation of the In:As ratio after ligand exchange.

We synthesized In(As, P) QDs by reacting InCl_3_ and DMA_3_As in oleylamine (OlNH_2_) using tris‐diethylaminophosphine as the reducing agent. As compared to published protocols,^[^ ^27^ ^]^ we increased the reaction temperature and changed the precursor concentrations to form different batches of QDs with average sizes of 5.0, 5.6, and 7.4 nm, see Experimental Section and Section S1, Supporting Information. With the use of powder X‐ray diffraction, we confirm the high degree of crystallinity of all three samples, see Section S2, Supporting Information. As shown in **Figure** **1**a, each batch exhibits a well‐defined band‐edge absorption feature at 1140, 1270, and 1400 nm, respectively. Hence, despite the minor admixing of phosphorous caused by the higher reaction temperature,^[^ ^30^ ^]^ the resulting In(As,P) QDs cover the blue part of the SWIR up to the wavelength range of 1400 − 1500  nm, which suffers little from background sunlight.

According to previous work,^[^ ^30^ ^]^ the In(As,P) QDs used here have a surface capped by a combination of chloride anions and OlNH_2_, where in particular OlNH_2_ will constitute a major barrier to charge transport. On the other hand, one equivalent of both surface moieties can be replaced as oleylammonium chloride ${\mathrm{OlNH}}_{3}^{+}{\mathrm{Cl}}^{-}$ by exposure to even weakly acidic compounds such as carboxylic acids or thiols.^[^ ^30^ ^]^ This insight inspired us to devise a liquid–liquid extraction process in which as‐synthesized In(As,P) QDs are transferred from apolar octane to polar dimethylformamide (DMF) using a mixture of 3‐mercapto‐1,2‐propanediol (MPD) and *n*‐butylamine (*n*‐BuNH_2_) as extracting agents. As shown in Figure 1b, this combination of MPD and *n*‐BuNH_2_ does induce a phase transfer of In(As, P) QDs from octane to DMF. Moreover, thiols can coordinate the In(As,P) QD surface after deprotonation and ${\mathrm{OlNH}}_{3}^{+}{\mathrm{Cl}}^{-}$ displacement, while *n*‐BuNH_2_ could lead to a direct exchange with surface‐bound OlNH_2_ or further promote the displacement of Cl^−^ through ${\mathrm{BuNH}}_{3}^{+}{\mathrm{Cl}}^{-}$ formation, see Figure 1c. Hence, apart from driving the phase transfer, the combination of MPD and *n*‐BuNH_2_ as extracting agents may induce a full removal of OlNH_2_ from the In(As,P) QD surface, a key step to enhance charge carrier mobility in In(As,P) QD films.

We investigated the post‐transfer In(As,P) QD surface chemistry through a combination of X‐ray photoelectron spectroscopy (XPS) and solution ^1^H nuclear magnetic resonance (NMR) spectroscopy. Quantitative NMR analysis indicates that after phase transfer and purification, at least 97% of the initial population of surface‐bound OlNH_2_ remains in the *n*‐octane phase. Furthermore, the NMR spectrum of the exchanged QDs in deuterated dimethylsulfoxide (DMSO‐*d*6) shows that both MPD and *n*‐BuNH_2_ are present in the final, purified polar dispersions (see Section S3, Supporting Information). Finally, XPS spectra recorded on phase‐transferred In(As,P) QDs show that the extraction process removes all surface‐bound chloride, while a sulfur signal appears at 162 keV – indicative of sulfide—and the In:As ratio remains unchanged, see Figure 1d. These observations are fully in line with the idea that the phase transfer comes with the full removal of surface‐bound OlNH_2_ and Cl^−^ from the In(As, P) QD surface, in exchange for the binding of BuNH_2_ and MPD as a thiolate. Moreover, we note that the XPS spectra give no evidence of As−S bond formation, nor of any surface oxidation during the phase transfer/ligand exchange process. The conclusion that the devised phase transfer procedure comes with a mere replacement of long‐chain by short‐chain ligands, without affecting stoichiometry nor inducing oxidation is further supported by the post‐transfer absorption spectra of In(As,P) QDs. As indicated in Figure 1a, these spectra coincide with pre‐transfer spectra, without noticeable shift of the band‐edge absorption feature and, for the 1270 and 1400 nm QDs, even a further narrowing of the absorption line.

**Figure 2:**
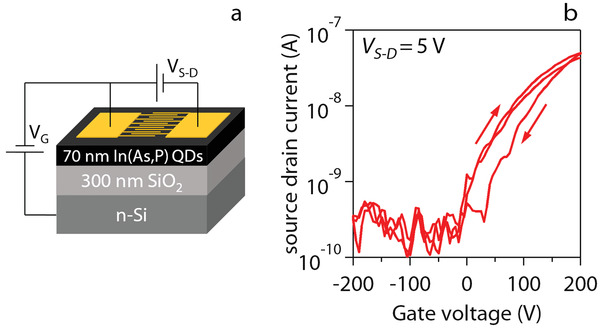
a) Schematic of the In(As, P) QD field effect transistor, consisting of a spincoated film of ligand exchanged QDs on top of cross‐fingered source and drain electrodes and separated from the gate electrode by a thermally grown oxide. b) Transfer characteristics of the field effect transistor at a source–drain voltage of 5 V.

Dispersions of post‐transfer In(As,P) QDs in DMF can be used to form In(As,P) QD films through spincoating. We assessed the semiconducting properties of such films through their integration in a field‐effect transistor (FET) consisting of interdigitated Au electrodes acting as source and drain contact and separated from an underlying silicon gate electrode by a 300 nm thick thermal oxide, see **Figure** **2**a. All handling—from phase extraction to FET formation and characterization—was executed in a nitrogen filled glovebox, and the In(As,P) QD film was gently annealed at 70 °C for 10 min prior to any measurements. Figure 2b displays the transfer (fixed *V*_SD_) curve recorded on a In(As,P) QD‐FET. As can be seen in Figure 2b, we observed a manifest conductivity increase for more positive gate bias, an indication of *n*‐type doping in the QD thin film. A similar conclusion has been put forward in the literature, regardless of the processing choices and synthesis chemistry.^[^ ^25^, ^28^, ^31^, ^32^, ^33^, ^34^, ^35^ ^]^ Depending on the direction of the gate‐voltage sweep, the range of measurable source–drain currents starts at gate voltages between 0 and 25 V, with noise‐level leakage currents at zero and negative gate bias. An ohmic regime is reached at $V_{G}\approx 5\;V$, from which we estimate a differential electron mobility of 1.4 10^−4^ cm^2^ V^−1^ s^−1^. Although QD thin films with mobilities multiple orders of magnitude higher have been demonstrated in the literature,^[^ ^36^ ^]^ we stress that this estimated number is an effective mobility under DC conditions that can suffice to create functioning photodiodes, as evidenced by reports on photovoltaic stacks based on halide and thiol‐passivated PbS QDs.^[^ ^37^, ^38^ ^]^

We confirmed the *n*‐type doping through UPS on a film of ligand‐exchanged In(As,P) QDs with an optical band‐edge transition at 1140 nm or 1.09 eV. As outlined in Section S4, Supporting Information, the photoelectron spectrum yields the energy of the valence‐band edge and the Fermi‐level as −5.03 and −4.41 eV, respectively. Using the optical band gap, we can position the conduction band at −3.94 eV, such that the Fermi level lies just above the middle of the band gap. This outcome suggests that the unipolar electron transport observed at positive gate bias is related to the presence of deep, mostly unionized donor levels in the QD film.

**Figure 3:**
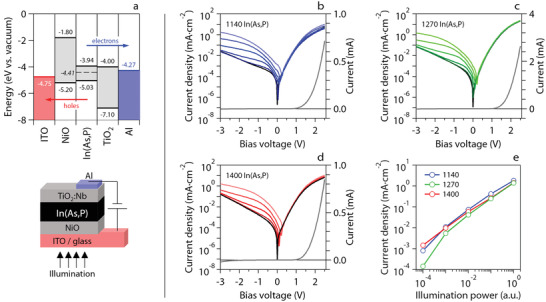
a) (top) Energy level diagram of the In(As,P) QDPD stack used here. The diagram was constructed by combining UPS results for the 1140 In(As,P) QD film and literature data for the contact materials.^[^ ^39^, ^40^, ^41^, ^42^, ^43^ ^]^ (bottom) Schematic of the QDPD stack. b–d) Dark and photocurrent densities under white‐light illumination of In(As,P) QDPDs for specific absorber layers as indicated. e) Photocurrent density as a function of white light illumination power in log–log scale. The reference power is 114.7 mW cm^−2^.

Given these insights, we sought to realize a rectifying junction that can function as a photodiode by depositing 120 nm thick *n*‐In(As,P) QD films on a *p*‐type NiO layer evaporated on ITO‐coated glass. NiO is a well‐known hole transport material in thin‐film photovoltaics,^[^ ^44^, ^45^, ^46^ ^]^ and was recently introduced to form photovoltaic stacks from *n*‐PbS QDs in a so‐called inverted structure with light incident at the *p*‐NiO|*n*‐QD junction.^[^ ^47^ ^]^ With valence‐band levels reported in literature around −5.20 eV and Fermi levels of −4.80 eV and lower, we expected *p*‐NiO to form a *pn* heterojunction with the *n*‐In(AsP) QD film.^[^ ^39^ ^]^ To improve rectification, we completed this stack through a spincoated electron transport layer consisting of Nb:TiO_2_ nanoparticles and an aluminum top contact. The layer formation sequence, where the *n*‐In(As,P) QD film deposition is preceded by NiO annealing at 300°C in air and followed by a mere gentle drying of the Nb:TiO_2_ nanoparticle film at 60°C under nitrogen, was designed so as to minimize the impact on the *n*‐In(As,P) QDs and extract holes through the NiO|ITO contact. **Figure** **3**a outlines the energy level diagram and structure of the implemented In(As,P) QDPD stack, indicating the polarity of the device where NiO and Nb:TiO_2_ block the flow of electrons and holes, respectively. Processing details are provided in the Experimental section and cross section scanning electron microscopy images in Section S5, Supporting Information.

We formed separate QDPD stacks with an active area of 0.13 cm^2^ using the three different In(As,P) QD batches included in this study. Indicating the different stacks as 1140, 1270, and 1400, in reference to the peak wavelength of the band‐edge absorption, Figure 3b–d represent their respective *j*–*V* characteristics. As highlighted by the linear representation of the dark current density, all three devices are clearly rectifying, with similar dark current densities of 0.85, 1.54, and 1.74 µA cm^−2^ at −1 V reverse bias. With increasing reverse bias, the difference in dark current density between the smallest and largest QDs increases, reaching almost one order of magnitude at −3 V. Note that reverse bias refers to the application of a negative voltage to the ITO contact, that is, a polarization meant to extract holes from the *n*‐In(As, P) QD film or inject electrons through the *p*‐NiO contact. We therefore conclude that the observed *j*–*V* rectification agrees with the intended stack structure.

**Figure 4:**
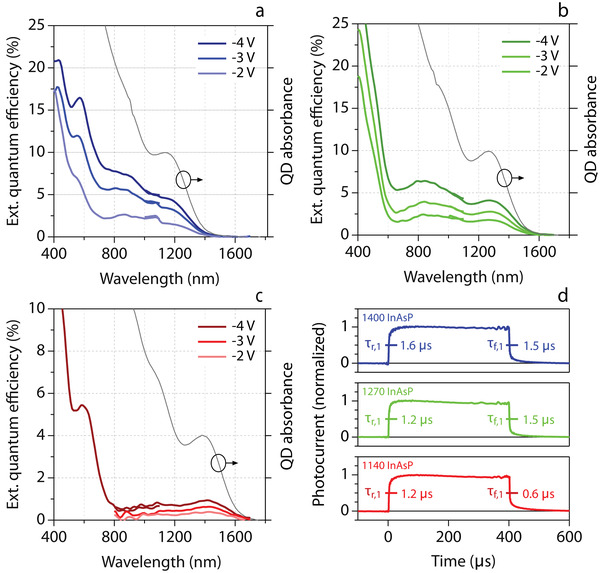
a–c) External quantum efficiency spectra for the different In(As,P) QDPDs as indicated, recorded at a reverse bias of −2, −3, and −4 V. The absorbance spectrum of the corresponding In(As,P) QD batch is added in each graph for comparison. d) Normalized transient photocurrent response of the different In(As,P) QDPDs following a 400 µs step illumination. Rise and fall times have been indicated by the dominant fast time constant obtained from a multi‐exponential fit of the transient.

Figure 3b–d also represents the photocurrent density recorded under illumination by a spectrally broad white LED at different power densities. As shown, we found that all devices featured a pronounced photoresponse at reverse bias. Moreover, photocurrent densities recorded at a reverse bias of −3 V scale approximately linearly when raising the illumination power by four orders of magnitude. As shown in **Figure** **4**a–c, we also observed that upon changing the wavelength of the excitation light from 1700 to 400 nm, the external quantum efficiency (EQE) of all three device stacks closely tracks the band‐edge absorption feature, peaking at 1150, 1270, and 1420 nm for the 1140, 1270, and 1400 In(As, P) QDPD stack, respectively. We thus conclude that the photoresponse of the *p*‐NiO|*n*‐In(As,P)|Nb:TiO_2_ stack is effectively due to light absorption in the In(As,P) QD film, and that the properties of the In(As,P) QDs are preserved throughout the QDPD fabrication.

Interestingly, whereas the 1400 QDPD stack only reached an EQE of 1% at 1400 nm, both the 1140 and 1270 stacks attain an EQE of around 5% at the band‐edge exciton transition and a reverse bias of −4 V. In fact, the 1270 QDPD stack yields a better EQE at 1400 nm than the 1400 stack – 2.7 versus 0.9%—a difference that is preserved when considering the internal quantum efficiency (IQE), where the EQE is corrected for the effective absorption of light by the QD layer in the stack. The calculated EQE spectra yield a responsivity at the band‐edge transition of 31, 29, and 7 mA W^−1^ for the 1140, 1270, and 1400 QDPDs, respectively. In a first approximation, these values can be used in combination with the dark current densities to estimate specific detectivities under the assumption that noise is dominated by shot noise, see Sections S7 and S8, Supporting Information. This approach yields specific detectivities of 1.110^10^, 6.110^9^, and 1.010^9^ cm Hz^0.5^ W^−1^ for the 1140, 1270, and 1400 QDPDs.

As shown in Section S6, Supporting Information, we estimate the IQE with the absorption of the full stack at the wavelength of maximized EQE at the respective band‐edge transitions, corrected for ITO absorption. The three QD layers absorb ≈10% of the incident light at the band‐edge transition when measuring reflection with the use of an integrating sphere. This results in an estimated IQE of 39±5% and 46±5% for the 1140 and 1270 stacks, respectively, while the 1400 stack only attains an IQE of 9±1% for the applied bias of −4 V. Possibly, the difference in IQE reflects a deteriorating band alignment between QDs with smaller band gaps and the charge‐transport layers, a point suggested by the energy‐band diagram shown in Figure 3a. In that case, the larger QDs in a polydisperse QD absorber can increasingly hamper charge transfer, an issue that will be most pronounced in the 1400 stack. On the other hand, we found that a switch to thicker films to absorb more light does little to improve the EQE, which indicates that charge carrier extraction limits the performance of each of the In(As,P) QDPD stacks studied here. This point is underscored by the considerably higher EQE of 15–30% at −4 V measured for 400 nm light, where the QD films have a significantly larger absorption coefficient and thus create more carriers close to the junction with NiO.

Last, we also investigated the rise and fall times of the three stacks in Figure 4d. The devices are placed under a constant reverse bias of −3 V and illuminated for 400 µs by a white LED. As shown in Section S9, Supporting Information, the rise and fall behavior can be analyzed as a multi‐exponential transient fit to the experimental data via chi‐squared minimization. The photocurrent rise is characterized by time constants in the range 1.2 − 1.6 and 8.9 − 18.1 µs, where the fast component contributes ≈75% of the eventual photocurrent. We accordingly indicated rise and fall times in Figure 4d through this dominant, fastest time constant. Ranging between 0.6 and 1.6 µs, these rise and fall times are comparable to some of the fastest mature PbS QDPDs, for which times of a few µs have been reported.^[^ ^48^, ^49^, ^50^ ^]^

## 3 Conclusion

In summary, we have demonstrated the formation of photodiodes for short‐wave infrared photodection based on In(As,P) QDs. These QDPDs were fabricated using QD dispersions in dimethylformamide, in which the original long‐chain organic ligands are replaced by short‐chain stabilizers. Following the observation that the resulting In(As,P) QD films exhibit *n*‐type doping, we introduce a QDPD stack based on a heterojunction between *n*‐In(As, P) QDs and *p*‐NiO on ITO, and using a film of Nb:TiO_2_ nanoparticles contacted by aluminum as the electron transport layer. Regardless of the QD band‐edge transition, which we vary from 1140 to 1270 and 1400 nm, these stacks show rectifying current–voltage behavior in the dark, with a reverse bias potential in agreement with the intended stack design, and exhibit a photocurrent under illumination that increases approximately proportional with the light intensity. For all devices, the external quantum efficiency tracks the QD absorbance spectrum, resulting in films photosensitive up to and beyond 1400 nm. While these first SWIR‐sensitive QDPD stacks based on non‐restricted In(As, P) QDs attain internal quantum efficiencies of up to 46%, further improvements are possible by enhancing the charge carrier extraction and improving the energy‐band alignment within the QDPD stack.

## 4 Experimental Section

### Materials

Oleylamine (OlNH_2_) >96% prim. amine, 80%–90% C18 content) was purchased from Acros Organics, dried over CaH_2_ and consequently vacuum distilled. Anhydrous and N_2_‐flushed toluene, ethanol, *n*‐octane and *N*,*N*‐dimethylformamide (DMF) in acroseal bottles were purchased from Acros Organics. Tris(diethylamino)phosphine (DEA_3_P, 97%), indium(III) chloride (>99.999%), *n*‐butylamine *n*‐BuNH_2_ and 3‐mercapto‐1,2‐propanediol (MPD) were purchased from Sigma‐Aldrich. *n*‐Butylamine was dried over CaH_2_, distilled, and flushed with N_2_ before use. Trisdimethylaminoarsine ((DMA_3_As), 99%) and tri‐*n*‐octylphosphine (minimum 97%) were ordered from Strem Chemicals.

### In(As,P) 1140 Synthesis

In a nitrogen filled glovebox, 1.4 mL of distilled OlNH_2_, 100 mg of InCl_3_ (0.45 mmol, 1 equiv.) and a stirring bar were loaded in a three‐neck flask. The flask was transferred from the glovebox to an inert gas Schlenk line and the mixture was degassed at 120°C for 1 h. In a separate vessel in the glovebox, 370 µL (3 equiv.) of DEA_3_P was transaminated with 1.5 mL OlNH_2_ by heating to 120°C under stirring until gas evolution stops. After degassing, the three‐neck flask containing InCl_3_ and OlNH_2_ was heated to 170°C under nitrogen and a mixture of 85 µL (1 equiv.) DMA_3_As and 500µL tri‐*n*‐octylphosphine was injected. After bubbling has terminated, the growth temperature was set at 270°C. During the temperature ramp, the transaminated aminophosphine reagent, cooled to room temperature, was injected into the reaction flask at 210°C . After injection of the aminophosphine, the reaction vessel was kept heating and attained 270°C in ≈180 s. The reaction was terminated by removing the heating mantle after 60 min of reaction time. Anhydrous, degassed toluene was added at 80°C, followed by purification through a precipitation and redispersion cycle using toluene and ethanol as solvent and non‐solvent. After a final redispersion in toluene, the QD dispersion was passed through a 0.450 µm pore size syringe filter.

### In(As,P) 1270 Synthesis

The synthesis procedure only differed in the concentration of reagents and the injection temperature. In the first step, 2.8 mL of distilled OlNH_2_ and 100 mg of InCl_3_ (0.45 mmol, 1 equiv.) were loaded with a stirring bar in a three‐neck flask. The injection of the transaminated aminophosphine precursor was done at 220°C. All other steps were identical to the above procedure.

### In(As,P) 1400 Synthesis

Same as above, except for a further dilution of the reaction mixture by dissolving 100 mg of InCl_3_ in 4.5 mL of OlNH_2_ in the first step. All other steps were identical.

### Liquid–Liquid Extraction and Ligand Exchange

50 mg of dry In(As, P) QDs capped with OlNH_2_ and Cl were dispersed in 2 mL of anhydrous *n*‐octane in the glovebox. In a separate vial, 2 mL of anhydrous and degassed DMF was mixed with 75 µL of distilled *n*‐BuNH_2_ and 150 µL of MPD. The QDs, dispersed in octane, were placed on top of the DMF phase by pipetting. After vigorously shaking the vial for 30 s, the two phases were separated through centrifugation. The QDs were hereby transferred to the DMF phase. The octane phase was removed and the DMF phase was washed three times with fresh *n*‐octane. The washed dispersion of QDs in DMF was precipitated by addition of 4 mL of toluene and centrifugation. The final pellet was dried under vacuum and redispersed in dry DMF and filtered prior to spincoating.

### XPS Characterization

Samples for X‐ray photoelectron spectroscopy (XPS) analysis were transferred using a dedicated transport vessel under high‐purity nitrogen atmosphere from the synthesis glovebox to an analysis glovebox attached to a vacuum line for surface analysis. In this analysis glovebox, the samples were taken out of the transport vessel and transferred into the XPS system without any exposure to ambient air. XPS measurements were performed on a Thermo Scientific Theta Probe instrument using monochromatic Al K‐alpha (1486.6 eV) radiation. The electron analyzer was set to a pass energy of 200 eV. The XPS data were analyzed with the CasaXPS software. Binding energies were calibrated against the In 4d peak at 17.43 eV.

### NMR Characterization

NMR measurements were recorded on a Bruker Avance III spectrometer operating at a ^1^H frequency of 500.13 MHz and equipped with a BBI‐Z probe. Quantitative ^1^H spectra were recorded with a 30 s delay between scans to allow full relaxation of the magnetization. All concentrations of surface‐bound ligands were determined according to the digital ERETIC method.

### UPS Measurements

(UPS measurements were carried out using a Physical Electronics PHI 5000 VersaProbe instrument. A monochromatized photon beam of 21.2 eV (He I) was utilized to acquire the spectra. The spectrometer was calibrated with Ag foil to determine the Fermi energy reference and a sample bias of −5 V was applied to obtain the secondary electron (SE) emission edge. Prior to record the spectra, sputter cleaning was performed on the samples using a cluster Ar beam at an energy per atom of 2.5 keV to remove surface contamination.

### FET Fabrication

Field‐effect tranistors were prepared by lithographically defined source and drain contacts evaporated on top of Si substrates with a 300 nm layer of thermally grown oxide. Ligand exchanged InAs QDs were spincoated onto the FET substrates at a concentration of 100 mg mL^−1^ and a spin speed of 850 rpm for 30 s, followed by a spinning cycle at 3000 rpm for 30 s. The acceleration of the spincoater was set to 150 rpm s^−1^. The spincoated film was dried on a hotplate at 60°C and stored in the glovebox overnight before measurement. Electrical measurements were performed with two Keithley 2400 source measuring units.

### QDPD Fabrication and Characterization

Indium tin oxide substrates were purchased. NiO was deposited via e‐beam evaporation and consequent annealing at 300°C in air prior to QD deposition. Ligand exchanged In(As, P) QDs were deposited on top of NiO by spincoating a QD solution at 200 mg mL^−1^ with the same spincoating program as in the FET fabrication. The film was annealed for 10 min at 60°C. After annealing and cooling down, Nb:TiO_2_ NPs were spincoated at a speed of 3000 rpm, followed by drying at 60°C under nitrogen. Top contacts were deposited via evaporation of aluminum through a shadow mask in a vacuum deposition system. QDPD *j*–*V* and transient photocurrent characterization was performed with a Paios system by Fluxim. The calibrated white light LED emitted a spectrum starting at 420 nm reaching across the visible and into the SWIR. Here, the maximum light intensity of 114.7 mW cm^−2^ was used as the illumination power reference. A custom setup consisting of a chopped QTH light source with monochromator, focusing optics, calibrated Si and Ge responsivity detectors, pre‐amplifier and lock‐in amplifier was used to measure external quantum efficiency as a function of wavelength.

### Statistics and Data Processing

All data analysis were performed using the Igor Pro Software package. Absorption traces in Figure 1 were normalized at the maximum of the band‐edge absorption feature and provided with an offset for clarity. Transient photocurrent traces in Figure 4d were normalized to the maximum photocurrent and the dark current was subtracted to yield a trace between 0 (dark current) and 1 (photocurrent) for all devices. All other data is presented as initially measured without processing. Average size determination and sizing statistics from transmission electron microscopy is discussed in Section S1, Supporting Information. Determination of rise and fall times from the photocurrent transients was performed with the fitting of a multi‐exponential model to the raw data. The goodness of fit was evaluated relatively for a single, double, or triple exponential model using an *F*‐test for regression.

## Conflict of Interest

The authors declare no conflict of interest.

## Supporting information

Supporting Information

## Data Availability

The data that support the findings of this study are available from the corresponding author upon reasonable request.
