# Supplementary material for: Colloidal III–V Quantum Dot Photodiodes for Short‐Wave Infrared Photodetection
Source: Adv Sci (Weinh). 2022 Apr 10;9(17):2200844. doi: 10.1002/advs.202200844 (PMC9189642; doi:10.1002/advs.202200844)
Supplement: Supplementary file 1 — Supporting Information [file ADVS-9-2200844-s001.pdf]

ADVANCED SCIENCE
Open Access

Supporting Information

for *Adv. Sci.*, DOI 10.1002/advs.202200844

Colloidal III–V Quantum Dot Photodiodes for Short-Wave Infrared Photodetection

*Jari Leemans, Vladimir Pejović, Epimitheas Georgitzikis, Matthias Minjauw, Abu Bakar Siddik, Yu-Hao Deng, Yinghuan Kuang, Gunther Roelkens, Christophe Detavernier, Itai Lieberman, Paweł E. Malinowski, David Cheyns and Zeger Hens**

Supporting Information For: Colloidal III-V Quantum Dot Photodiodes for Short-Wave Infrared Photodetection

Jari Leemans,[†] Vladimir Pejović,[‡] Epimitheas Georgitzikis,[‡] Matthias Minjauw,[¶] Abu Bakkar Siddik,[‡] Yu-Hao Deng,[†] Yinghuan Kuang,[‡] Gunther Roelkens,[§] Christophe Detavernier,[¶] Itai Lieberman,[‡] Paweł E. Malinowski,[‡] David Cheyns,[‡] and Zeger Hens[*,†]

†*Physics and Chemistry of Nanostructures, Ghent University, 9000 Gent, Belgium*

‡*Imec vzw, 3001 Leuven, Belgium*

¶*Department of Solid State Science, Ghent University, 9000-Gent, Belgium*

§*Photonics Research Group, 9052 Gent, Belgium*

E-mail: zeger.hens@ugent.be

S1 Transmission Electron Microscopy

To determine the size of the respective QD samples, ensemble transmission electron microscopy images were recorded by dropcasting dilute solutions of the QDs on a TEM grid. The images for the QDs absorbing at 1140, 1270 and 1400 nm can be seen in Figure S1a, b and c respectively. Size analysis of the TEM images yields average particle sizes of 5.0, 5.6 and 7.4 nm, taken as the height of the projected triangles. The respective standard deviations σ of the QD size absorbing at 1140, 1270 and 1400 nm are 0.5, 0.5 and 0.6 nm respectively. Average particle sizes are calculated as the arithmetic mean, standard deviations

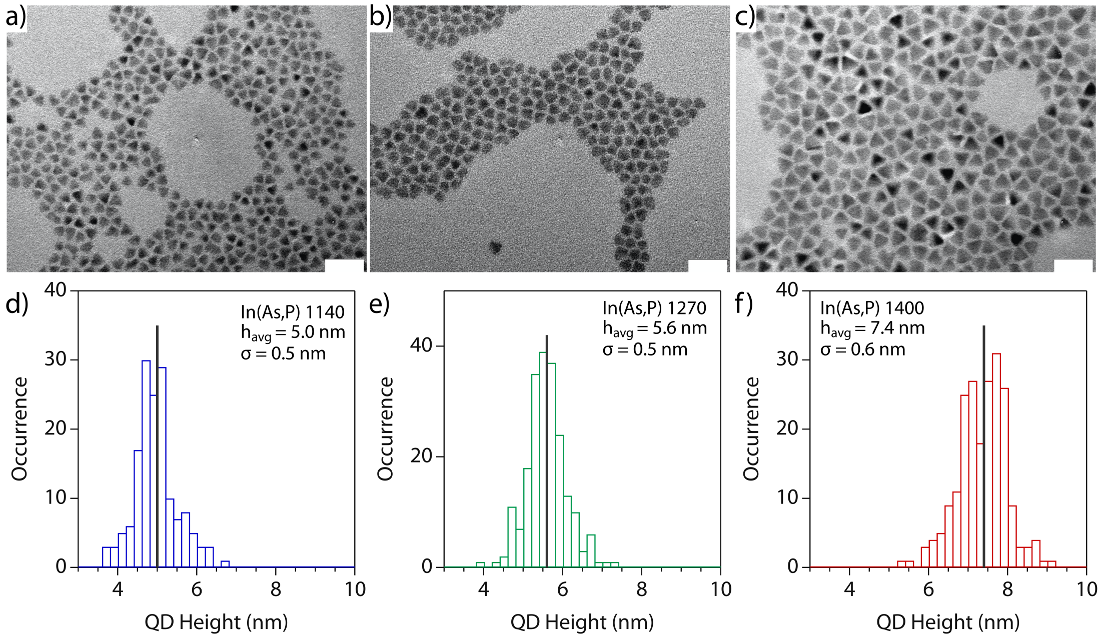

Figure S1: Ensemble TEM images for QDs with an excitonic absorption feature at a) 1140 b) 1270 and c) 1400 nm. All scale bars correspond to 20 nm. Average sizes are extracted from measuring the height of individual nanocrystals in the projected images. Histograms of the sizing data are provided for the d) 1140, e) 1270 and f) 1400 samples. Black line indicates the arithmetic mean and annotated standard deviation is calculated from the raw data.

are also calculated from the raw data. The sizing data is sampled from 155-210 individual QDs and plotted in the form of histograms in Figure S1d-e with a bin width of 0.2 nm. The three samples all show population distributions centered around the mean value.

S2 X-ray diffraction analysis

In order to evaluate the crystallinity of the three QD samples, we performed powder x-ray diffraction measurements and the respective diffractograms are presented here in Figure S2a-c for the 1140, 1270 and 1400 samples. The diffraction data indicates the expected cubic reflections which are annotated atop the diffractograms for the In(As,P) alloys. To verify that the different samples have a similar lattice constant, we compared the diffraction angles after extracting them with a peak fit to the (220) and (311) reflections, as they are isolated

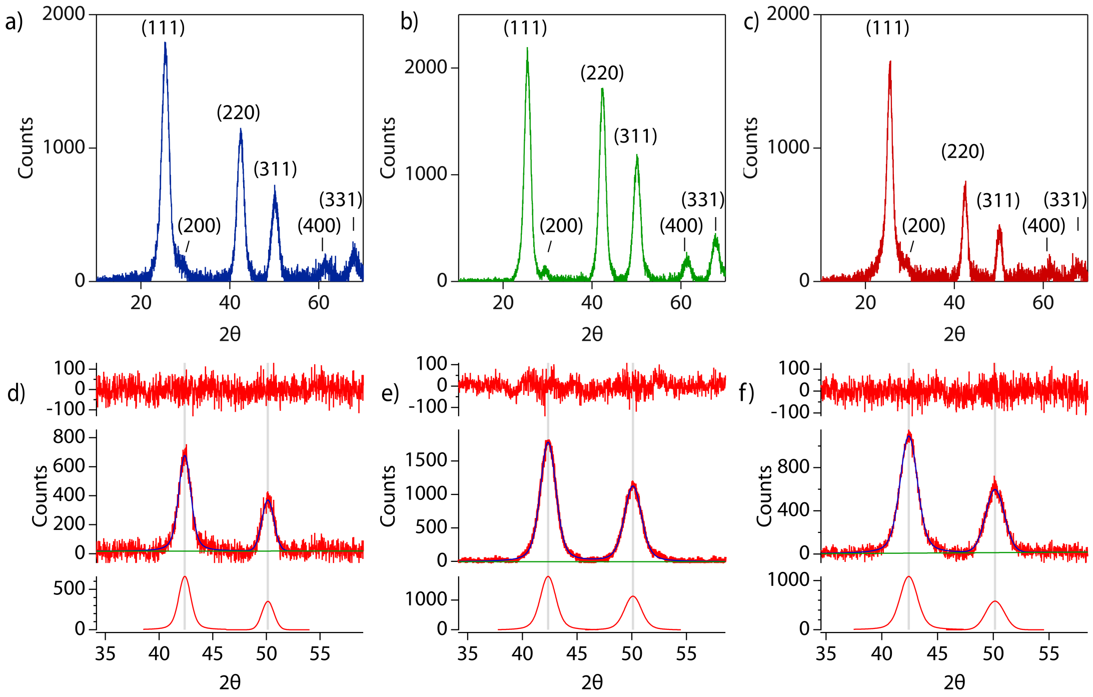

Figure S2: Powder x-ray diffractograms of the a) 1140, b) 1270 and c) 1400 QD samples. Peak fits used to extract the peak position of the (220) and (311) reflections illustrated on top of the diffractograms for the d) 1140, e) 1270 and f) 1400 samples.

and present a good signal to noise ratio for interpretation. The peak fits are visualized in Figure S2d-f and the extracted peak data is summarized in Table S1. The differences in the diffraction angles suggests at most a difference in interplanar distance d_{hkl} between the samples of 0.1% as determined according to Bragg's Law. This analysis confirms that the lattice constant of the 3 samples is highly similar.

To obtain an estimate of whether the nanocrystals are single crystals or polycrystalline,

Table S1: XRD data extracted from the diffractograms of the three QD samples used in the QDPDs of the main manuscript.

Sample	Peak (hkl)	2θ	FWHM	Scherrer size (nm)	TEM size (nm)
1140	(220)	42.41±0.01	0.0303	6.2	5.0±0.5
1140	(311)	50.15±0.01	0.0321	6.7	5.0±0.5
1270	(220)	42.35±0.01	0.0275	6.8	5.6±0.5
1270	(311)	50.10±0.01	0.0300	7.2	5.6±0.5
1400	(220)	42.40±0.01	0.0215	8.7	7.4±0.6
1400	(311)	50.14±0.01	0.0206	10.5	7.4±0.6

an estimate of the crystallite size via the Scherrer equation can be compared to the size obtained from transmission electron microscopy (TEM). The averaged values obtained for the crystallite size after applying the Scherrer equation to the instrument corrected full-width at half maximum (FWHM) of the (220) and (311) reflections are 6.2 and 6.7 nm for the 1140 sample, 6.8 and 7.2 nm for the 1270 sample and 8.7 and 10.5 nm for the 1400 sample. The Scherrer sizes match roughly to the respective average dimensions of 5.0±0.5, 5.6±0.5 and 7.4±0.6 nm from TEM. Although a slight overestimation of the crystallite size is obtained with the Scherrer equation, the data suggests that the nanocrystals are single crystalline.

S3 ^{1}H-NMR Characterization of Ligand Exchange

To investigate whether the liquid-liquid extraction induced by 3-mercapto-1,2-propanediol (MPD) and n-butylamine in dimethylformamide (DMF) efficiently replaces the native $OlNH_2$ from the QD surface, ^{1}H-NMR spectra were recorded on a replica experiment with In(As,P) QDs synthesized according to the same procedures from the main manuscript. QDs were dispersed first in toluene-d_8 to record the 1D ^{1}H-NMR spectrum of the purified sample. The spectrum is presented in Figure S3a. The characteristic spectrum of $OlNH_2$-capped QDs is retrieved, which is extensively described in a recent surface chemistry report.[1] With the digital ERETIC method, the concentration of the ligands was measured to be 26.7 mmol.L^-1. After drying the sample, the QDs are dispersed in n-octane for the 2-phase ligand exchange to DMF. The transparent n-octane phase is collected after extraction and dried. The spectrum of the dreid n-octane phase is presented in Figure S3c, resonances characteristic of freely dissolved $OlNH_2$ at a concentration of 25.9 mmol.L^{-1} are identified. The extracted QDs in dimethylformamide are precipitated twice by addition of deuterated toluene, after which they are finally redispersed in deuterated dimethylsulfoxide (DMSO-d_6). The spectrum of the exchanged QDs is presented in Figure S3b alongside the resonances of the extractants n-$BuNH_2$ and MPD.

Importantly, quantitative analysis of the n-octane fraction in Figure S3c suggests that 97% of the $OlNH_2$ bound to the surface of the QDs is removed during the 2-phase ligand exchange already before the precipitation steps. This proves that the extracted QDs undergo ligand exchange during extraction. The spectrum of the exchanged QDs features sharper resonances lacking fine structure which correspond to the spectra of the small molecule extractants on top of a broad pedestal. The presence of narrow and broad peaks can be attributed to surface bound MPD and n-$BuNH_2$ alongside a minor excess which might contribute additionally to the stability of the dispersion. This interpretation is validated by the analysis of the DOSY decay trace in Figure S3d, demonstrating that the small molecules diffuse at a speed corresponding to the QD size, whereas a sizeable subfraction diffuses at a considerably faster rate. Importantly, also the fast diffusion constant corresponds roughly to 1 nm species in solution, much larger than the size of n-$BuNH_2$ and MPD, indicating that also this more quickly diffusing subfraction interacts with the QD surface.

The ^{1}H-NMR study demonstrates conclusively that the extraction to the DMF phase is facilitated by ligand exchange with the small molecule extractants. A near unity removal of $OlNH_2$ is achieved with trace amounts remaining in the polar phase.

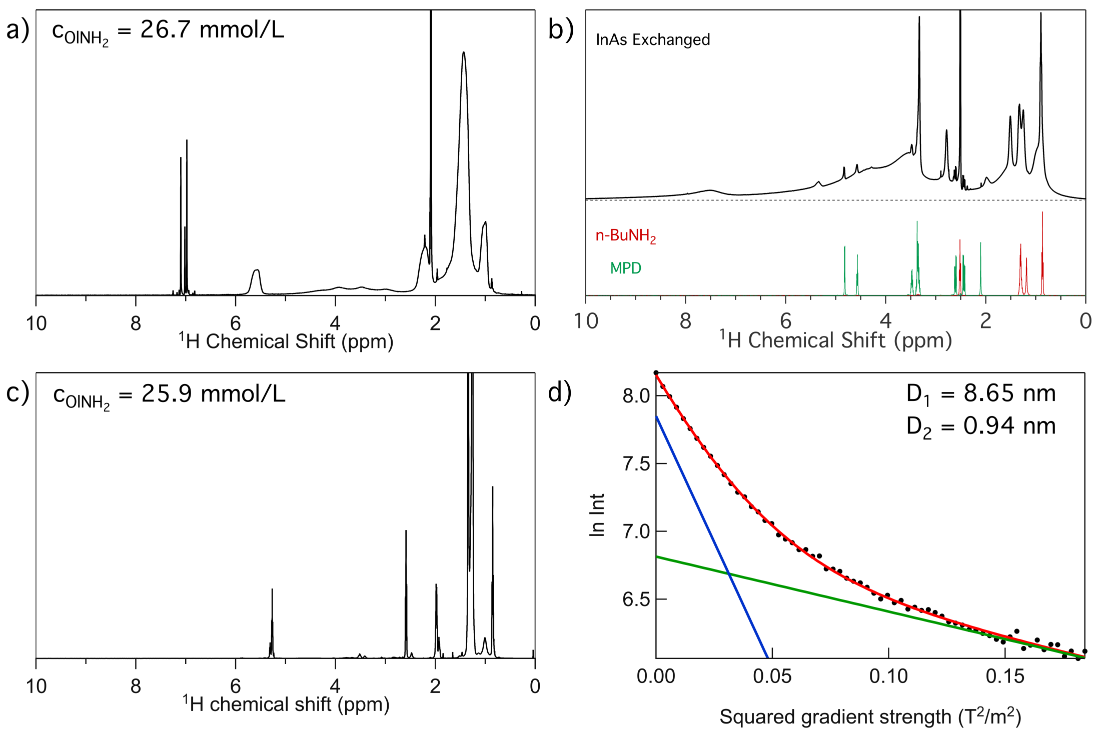

Figure S3: ^{1}H-NMR spectra of a) In(As,P) QDs passivated by $OlNH_2$ in toluene-d8, b) ligand-exchanged and extracted QDs in DMSO-d6, c) the collected n-octane fraction after ligand exchange dissolved in DMSO-d6. d) The DOSY decay trace of the ligand exchanged In(As,P) QDs in DMSO-d6 indicates the presence of surface bound ligands as well as a subfraction which is most likely in a dynamic bound-unbound state on the QD surface.

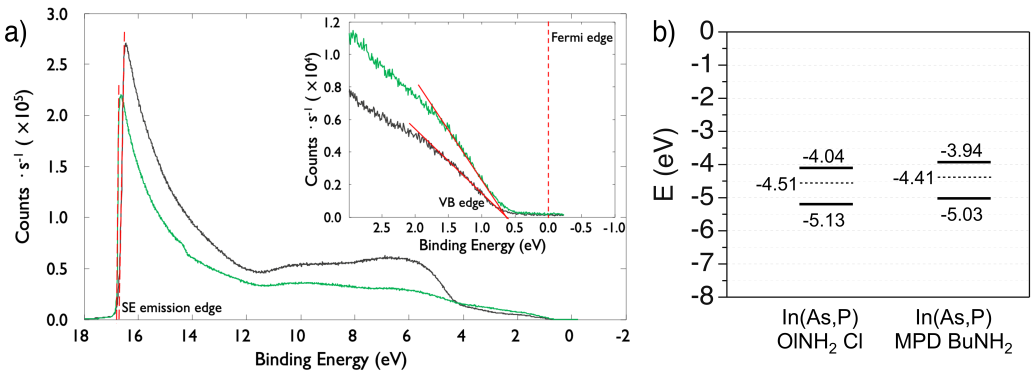

Figure S4: a) Ultraviolet photoelectron spectra of native (black) and ligand exchanged (green) In(As,P) QDs and b) their respective energy levels as extracted from the UPS spectra in combination with the optical band gap.

S4 Ultraviolet Photoelectron Spectroscopy

Ultraviolet photoelectron spectroscopy was performed on both a spincoated sample of ligand exchanged and native $OlNH_2$ and chloride-capped In(As, P) QDs on ITO-coated glass. The full spectra are provided here in Figure S4a and the extracted energy level with respect to vacuum are presented in Figure S4b. To position the conduction band, the optical band gap was taken at the peak maximum of the band-edge transition in absorption spectroscopy. Both samples are slightly n-doped, with a fermi level lying above the middle of the band gap. Interestingly, rather than affecting the doping, UPS results suggest that the developed ligand exchange chemistry only has an effect on the absolute position of the energy levels with respect to vacuum.

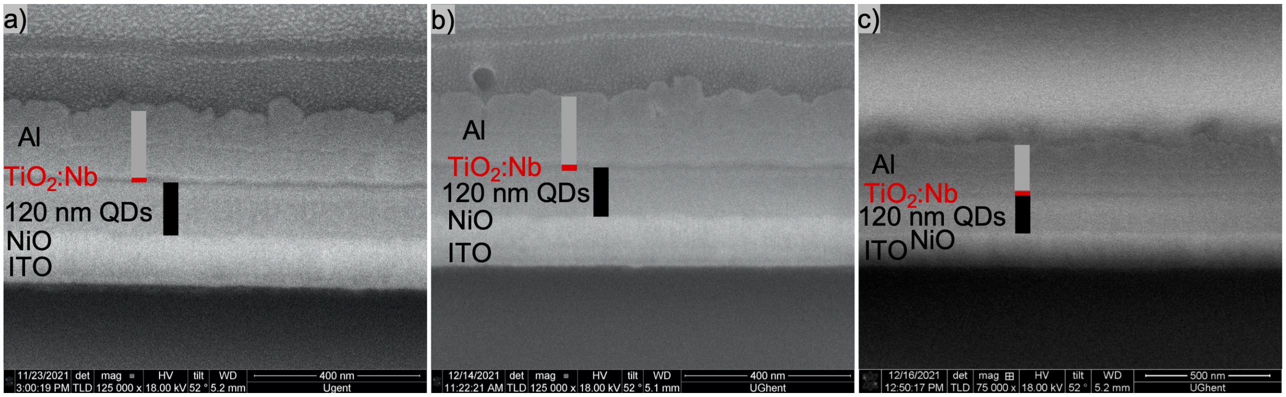

Figure S5: Scanning electron microscope cross section images of the QDPDs based on In(As, P) QDs with a band-edge absorption feature centered at a) 1140, b) 1270 and c) 1400 nm. The QD layer thicknesses are approximately 120 nm thick in each device.

S5 Cross-Section SEM of the In(As,P) QDPDs.

The thickness of the QD layer in the photodiode stacks was extracted from cross section scanning electron microscopy images. The images for the stacks absorbing at 1140 nm, 1270 nm and 1400 nm are presented in Figure S5a, b and c, respectively. The QD layers are all approximately 120 nm in thickness. The thickness of the Nb-doped TiO_2 layer amounts to roughly 15 nm, whereas the thickness of the NiO is impossible to estimate from these images due to insufficient contrast.

S6 Absorption and Internal Quantum Efficiency of the In(As,P) QDPDs

To evaluate the external quantum efficiency results presented in Figure S6a-c and in the main manuscript it is illustrative to investigate the actual absorption of light in the photodiode stacks. The absorption spectra of the three stacks measured in reflection mode is presented in Figure S6d, e and f respectively. The excitonic absorption features of the 3 samples are identified and match the absorption of the same samples in solution. Furthermore, only roughly 10 percent of incident light is effectively absorbed by the QD layer at wavelengths corresponding to the respective band-edge transitions. A large background absorption from the indium tin oxide contact causes the absorption to increase to nearly 30% at the respective band-edge transitions. We then define the internal quantum efficiency (IQE) by dividing the external quantum efficiency by the percentage of light that is absorbed by the QD film in the stack. To estimate the absorption by the QDs, we subtract the absorption of ITO on the low-energy side of the band-edge transition from the absorption at the wavelength of the band-edge feature where EQE is maximized. The IQE of the 1140, 1270 and 1400 stacks equal $39 \pm 5\%$, $46 \pm 5\%$ and $9 \pm 1\%$ at the respective band-edge absorption maxima and 4 V reverse bias. The error is estimated based on the error made when choosing a background to extract the absorption in the QD film as is indicated with the black lines on the absorption spectra in Figures S6d-f.

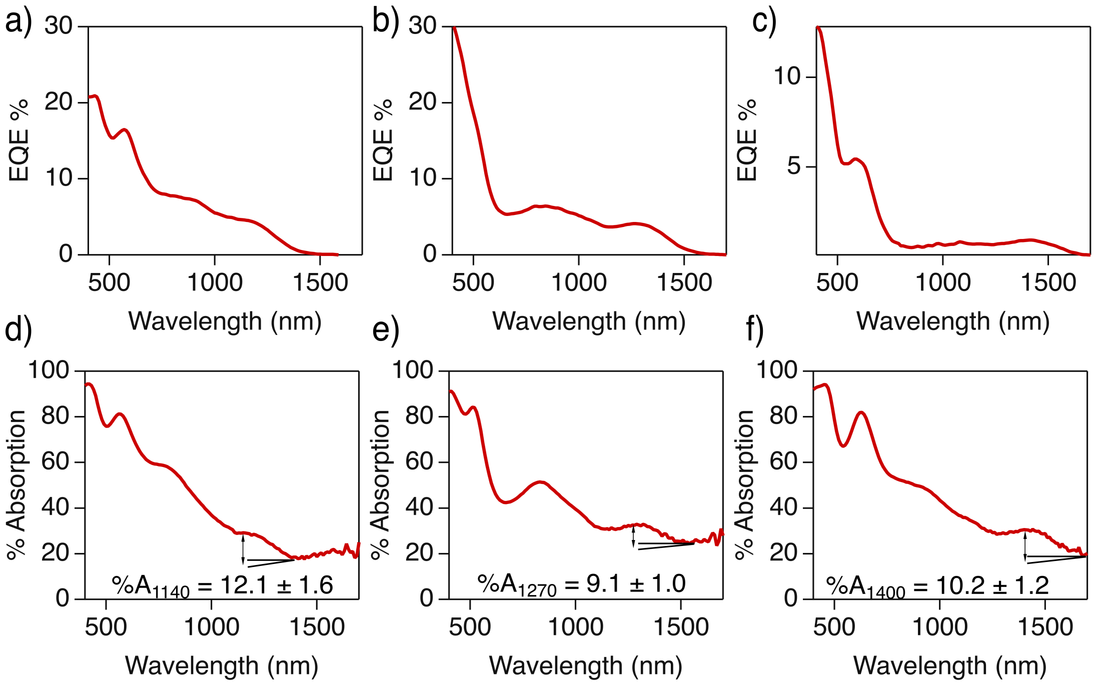

Figure S6: External quantum efficiency of the a) 1140, b) 1270 and c) 1400 QDPDs. Full stack absorption as measured in reflection mode for the d) 1140, e) 1270 and f) 1400 QDPDs. Annotations indicate the estimated absorption by the QD film in the full stacks as is needed to determine the internal quantum efficiency at the respective band-edge transitions.

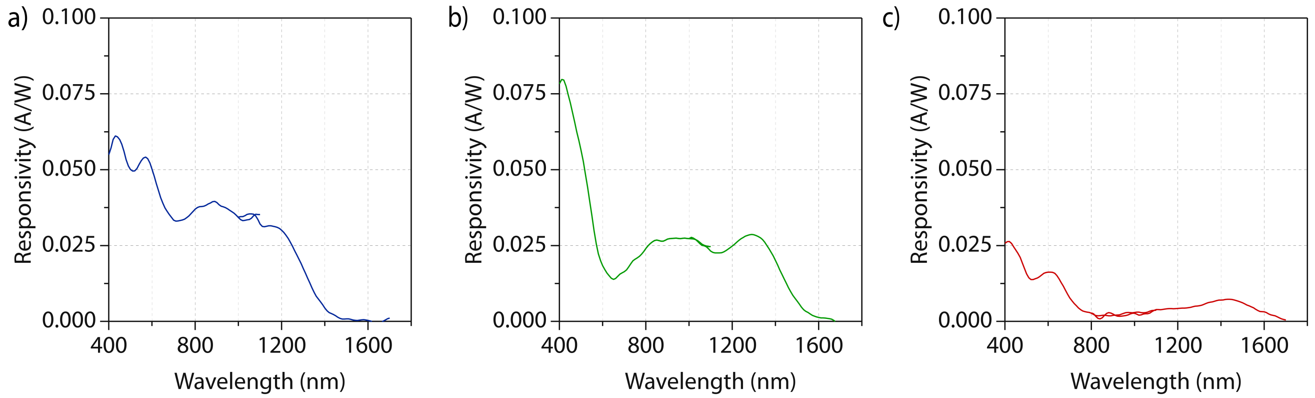

Figure S7: Responsivity of the a) 1140, b) 1270 and c) 1400 QDPDs from the main manuscript. The responsivity can be easily calculated from the external quantum efficiency discussed in the main text.

S7 Responsivity of the In(As,P) QDPDs

The responsivity of the photodiodes has been calculated at every wavelength from the external quantum efficiency plots in Figure 4 of the main manuscript according to Equation 1. The respective plots have been included here in Figure S7a-c. The 1140, 1270 and 1400 photodiodes reach responsivities at the maximum of their respective band-edge transitions and 3 V reverse bias of 0.031, 0.029 and 0.007 A/W.

$$R(\lambda) = EQE(\lambda)\frac{e\lambda}{hc} \tag{1}$$

S8 Estimated Specific Detectivity of the In(As,P) QDPDs

A first approximation of specific detectivity can be made assuming that the shot noise in the photodiodes is limiting, as is the case in previously published QDPDs based on PbS, where only at low frequencies 1/f noise appears.[2,3] Under such circumstances, the spectral noise density can be calculated from the dark current as in Equation 2 and the specific

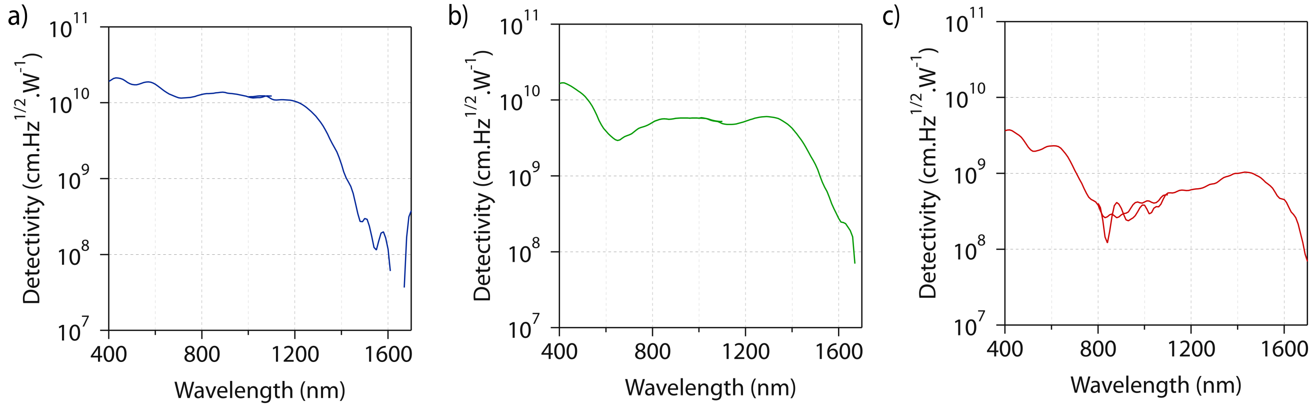

Figure S8: The approximated specific detectivity D* from the calculated responsivity and the assumption that shot-noise is limiting the device noise. The calculated D* is plotted throughout the visible and SWIR for the a) 1140, b) 1270 and c) 1400 QDPDs.

detectivity follows with Equation 3. Following this approximation, we present the plots of specific detectivity as a function of wavelength in Figure S8a-c for the 1140, 1270 and 1400 QDPDs. The 1140, 1270 and 1400 QDPDs reach detectivities at their band-edge transitions of $1.1 * 10^{10}$, $6.1 * 10^{9}$ and $1.0 * 10^{9}$ cm.$Hz^{1/2}$.W^{-1} respectively. Following the same approximation of shot-noise limitation, noise equivalent power was estimated for the three devices at 42, 306 and 706 μW.

$$S = \sqrt{2eI_d} \tag{2}$$

$$D^*(\lambda) = \frac{R(\lambda)}{\sqrt{2ej_D}} \tag{3}$$

S9 Photocurrent Transients

During the transient photocurrent experiment, the rise and fall of the photocurrent at constant bias are obtained during turning on and off the LED, respectively. To interpret the data from this experiment, we fit the exponential rise and fall of the photocurrent plotted in Figure S9 a and b with a multi-exponential as described by Equation 4. The current rise

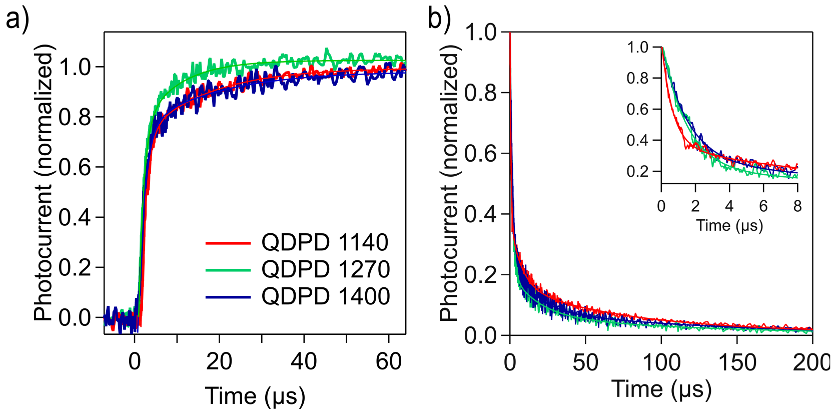

Figure S9: a) Rise and b) fall of the transient photocurrent. The traces are isolated from the transient photocurrent graph presented in the main manuscript. The respective multi-exponential fits are plotted atop the raw data.

is well modelled with two exponential components (n=2) differing approximately 1 order in magnitude, whereas a third component is introduced for the photocurrent decay. The fitting is performed in the Igor Pro software according to a chi-squared minimization. The time constants and pre-exponential factors derived from the fits are summarized in Table S2.

Importantly, both in the case of the rise and fall of the photocurrent, approximately 75% of the transient magnitude is described by the fastest component τ_1. For all three diodes, the fastest time constant lies between 0.6 and 1.6 microseconds, whereas the second components are approximately 1 order of magnitude slower.

$$I = I_0 + \sum_{i=1}^{n} A_i * exp\left(\frac{-t}{\tau_i}\right) \tag{4}$$

Table S2: Fitting constants obtained from fitting the normalized photocurrent rise and fall to a multi-exponential function.

Photocurrent rise	QDPD 1140	QDPD 1270	QDPD 1400
I_0	0.97	1.03	0.98
A_1	-0.76	-0.75	-0.81
τ_1 (μs)	1.24	1.18	1.55
A_2	-0.27	-0.28	-0.23
τ_2 (μs)	15.6	8.90	18.1
Photocurrent fall			
I_0	0	0	0
A_1	0.66	0.84	0.75
τ_1 (μs)	0.63	1.46	1.53
A_2	0.22	0.15	0.18
τ_2 (μs)	7.45	18.6	14.7
A_3	0.16	0.06	0.09
τ_3 (μs)	78.0	146.2	129.6

References

(1) Leemans, J.; Dümbgen, K. C.; Minjauw, M. M.; Zhao, Q.; Vantomme, A.; Infante, I.; Detavernier, C.; Hens, Z. *J. Am. Chem. Soc.* **2021**,

(2) Pal, B. N.; Robel, I.; Mohite, A.; Laocharoensuk, R.; Werder, D. J.; Klimov, V. I. *Adv. Funct. Mater.* **2012**, *22*, 1741–1748.

(3) Manders, J. R.; Lai, T.-H.; An, Y.; Xu, W.; Lee, J.; Kim, D. Y.; Bosman, G.; So, F. *Adv. Funct. Mater.* **2014**, *24*, 7205–7210.
